# Radioiodination of 2,3-dimethyl-4*H*-furo[3,2-*c*]coumarin and biological evaluation in solid tumor bearing mice

**DOI:** 10.1016/j.apradiso.2014.09.011

**Published:** 2015-01

**Authors:** S.M. Abd Elhalim, I.T. Ibrahim

**Affiliations:** Labelled Compounds Department, Radioisotopes Production and Radioactive Sources Division, Hot Laboratories Center, Atomic Energy Authority, P.O. Box 13759, Cairo, Egypt

**Keywords:** Coumarin, Iodine, Labeling, Biodistribution, Solid tumor

## Abstract

Compound 2,3-dimethyl-4*H*-furo[3,2-*c*]coumarin is a coumarin derivative that could be labeled with ^125^I. The process of labeling was started using 1 mg of the compound, 50 µg CAT oxidizing agent, 30 min as reaction time at pH with a yield about 95%. The ^125^I-coumarin derivative was stable for about 48 h. Radiochemical purity of the labeled compound was performed by electrophoresis and HPLC. The labeled compound was separated with purity about 95%. Tumor transplantation to produce a solid tumor in the right leg of albino mice was made by intramuscular injection of 2×10^6^ EAC (Ehrlish acittes carcinoma cells). Biodistribution study of ^125^I-coumarin derivative revealed that the uptake in tumor bearing leg was over 5% at 1 h and 4 h post-injection. This uptake encourages the use of ^123^I-coumarin derivative in imaging of tumor sites.

## Highlights

• Labeling of coumarin derivative with iodine-125.
• The appropriate conditions for labeling of coumarin derivative (95% yield).
• ^125^I-coumarin derivative was found to be highly localized in tumor.

## 1 Introduction

Early diagnosis of a tumor is still one of the most significant problems to start treatment before metastasis. One of the routes to tumor imaging is to label anticancer drugs (antimetabolites, alkylating agents, metabolites, peptides, hormones and DNA or RNA interacting agents like quinoxalines and coumarins) (Adelstein and Kassis, 1996).

Coumarin is a chemical compound which is found naturally in some plants, although it can be synthetically produced as well. It has a distinctive odor which has led people to use it as a food additive and ingredient in perfume. The chemical name for coumarin is benzopyrone. Coumarins are structural units of several natural products and feature widely in pharmacologically and biologically active compounds (O’Kennedy and Thornes, 1997). Coumarins have been synthesized by several routes including von Pechmann, Perkin, Knoevenagel, Reformatsky and Wittig reactions (von Pechmann and Duisberg, 1884, Johnson, 1942, Jones, 1967, Brufola et al., 1996, Shriner, 1942, Yavari et al., 1998). Besides functionalized coumarins furocoumarins such as psoralens are photoactive drugs which are extensively used in the PUVA (psoralen plus UVA radiation) therapy for the treatment of human skin diseases (Singer and Long, 1996, Zahradnik, 1992, Murray et al., 1982). Most prominent among the biological activities associated with these photochemotherapeutic agents (psoralens, linear furocoumarins) is their ability to cross-link DNA *via* intercalation of the furocoumarin between the base pairs of the nucleic acid and [2+2] photocyclo addition with the pyrimidine bases, particularly thymine (Maria et al., 2004). Based on their interaction with DNA the idea was to label one of these compounds (Hara et al., 2001).with radioiodine to image tumor sites. Synthesis of 2,3-dimethyl-4*H*-furo[3,2-*c*] coumarin and 3-phenyl-4*H*-furo[3,2-*c*] coumarin as angular furocoumarins were carried out through the Williamson reaction of 4-hydroxycoumarin with α-haloketones followed by cyclization (Jiao et al., 2003).

The affinity of 2,3-dimethyl-4*H*-furo[3,2-*c*] coumarin towards DNA and the antibacterial activity were evaluated and compared with 8-methoxypsoralen (8-MOP) (Dall’Acqua et al., 1981).

**Fig. 1:**
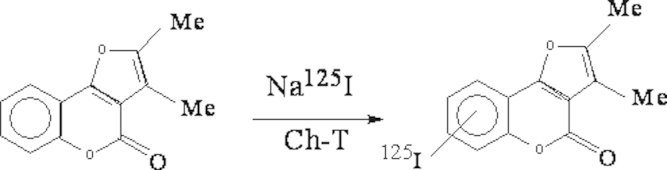
Labeling of coumarin derivative with radioiodine [^125^I] using CAT as oxidizing agent.

This study was conducted to label 2,3-dimethyl-4*H*-furo[3,2-*c*] coumarin with radioiodine [^125^I] (Fig. 1) and study factors affecting labeling yields. It will be extended to evaluate the radiolabeled coumarin derivative with convenient chromatographic methods. The study also searches the ability of the [^125^I-compounds] to imaged an induced solid tumor in EAC bearing mice.

## 2 Materials and methods

### 2.1 Drugs and chemicals

1. Iodine-125 was purchased from Institute of Isotope Production, Belgum.
2. 2,3-dimethyl-4*H*-furo[3,2-*c*]coumarin is a gift from Faculty of Science-Damietta University, New Damietta.
3. Chloramine-T was purchased from Sigma Chemical Company, USA.
4. All other chemical reagents were of analytical grade (AR), obtained from reputable manufacturers.
5. Ehrlich ascites carcinoma (EAC) was kindly supplied from National Cancer Institute, Cairo, Egypt.

Female Swiss Albino mice weighing 20–25 g were purchased from the Institute of Eye Research Cairo, Egypt. Environmental and nutritional conditions were kept constant throughout the experimental period and kept at room temperature (22±2) °C with a 12 h on/off light schedule. Female mice were used in this study due to their susceptibility to Ehrlich ascites carcinoma more than male mice (Wally et al., 2010). Animals were kept with free access to food and water throughout the experiment.

Synthesis of 2,3-dimethyl-4*H*-furo[3,2-*c*]coumarin and 3-phenyl-4*H*-furo[3,2-*c*] coumarin. The synthetic route for the preparation of the furocoumarin derivative is displayed as phenol was converted in 50% yield to the 4-hydroxycoumarin by the reaction with malonic acid in the presence of phosphorous oxychloride as described in the literature by Al-Sehemi and El-Gogary (2012). Synthetic compound was evaluated by IR, NMR and mass spectroscopy. The structure of the compound was characterized by IR, 1H NMR, 13C NMR, mass spectroscopy and elemental analysis.

### 2.2 Iodine labeling

^125^I-coumarin derivative was prepared by the following procedure: 10 mg coumarin derivative was dissolved in 1 ml DMF with stirring. CAT solution was prepared as 10:1 (w/w) in double distilled water then about 5–10 µL (50–100 µg CAT) was added to 100 µl coumarin derivative solution in dark colored tube and approximately 50–100 MBq ^125^I at room temperature. After a specified interval of time, the reaction was stopped using 0.2 N Na_2_S_2_O_3_ solutions to ensure that the un-reacted iodine is reduced before chromatographic analysis (Kassis et al., 2004). The yield of the reaction and the radiochemical purity were determined by paper electrophoresis. Paper electrophoresis techniques were conducted to distinguish between free iodide that move towards the anode at the top, while iodocompound persist near the point of spotting.

## 3 Factors affecting labeling yield

There are many factors that affect labeling yield such as follows:

### 3.1 Oxidizing agent

To conduct this experiment different amounts of CAT (10, 25, 50. 100, 150 and 200 µg) were used to obtain the optimum one.

### 3.2 Substrate content

To investigate the minimum amount at which maximum labeling yield was obtained, different amounts of coumarin derivative (100, 300, 500, 1000, 1500 and 2000 µg) were used.

### 3.3 pH of the reaction

The pH of the medium is one of the important factors that affect labeling yield. So the reaction medium was studied at different pH values (2, 4, 7, 9, 11) using pH Meter model 601A digital ionalyzer, Orion research, USA to distinguish the pH at which maximum labeling yield was obtained.

### 3.4 Reaction time

Labeling reactions proceeded to different reaction times (1, 5, 10, 15, 20, 25, 30, 45 and 60 min).

### 3.5 Reaction temperature

The reaction was carried out at different temperatures 25, 40, 50 and 70 °C.

### 3.6 *In-vitro* stability

Stability was studied to determine the stability of ^125^I-coumarin derivative after labeling and the impact of time on that compound. The yield was measured at different time intervals (1, 4, 12, 24 and 48 h) after labeling. When one factor was studied other factors were kept constant.

### 3.7 Electrophoresis conditions

Electrophoresis was done with EC 3000 p-series 90 programmable power and chamber supply units using cellulose acetate strips (45 cm). These stripes were moistened with 0.05 M phosphate buffer pH 7 and then introduced in the chamber. Samples were applied at a distance of 10 cm from the cathode. Standing time and applied voltage were continued for one and a half-hours. Developed strips were dried and cut into 1 cm segments, then counted by a well-type NaI scintillation counter. The radiochemical yield is calculated as the ratio of the radioactivity of the labeled product to the total radioactivity (Kassis, 2003).

$$\%\;\mathrm{Radiochemical}\;\mathrm{yield}=\frac{\mathrm{Peak}\;\mathrm{activity}\;{\mathrm{of}}^{125}I-\text{coumarin}\;\mathrm{derivative}}{\mathrm{Total}\;\mathrm{activity}}\times 100$$

### 3.8 Tumor transplantation in mice

Ehrlich ascites carcinoma cells (EAC) is a model for studying the biological behavior of malignant tumors and drugs assumed to produce effects at these sites (Ibrahim and Wally, 2010).

A line of Ehrlich ascites carcinoma (EAC) was maintained in female Swiss Albino mice through weekly I.P transplantation of 2.5×10^6^ tumor cells/mouse. EAC cells were obtained by needle aspiration with aseptic condition. The ascetic fluid was diluted with sterile saline so that 0.1 ml contains 2.5×10^6^ cells counted microscopically using a hemocytometer. About 0.2 ml solution was then injected intramuscularly in the right leg to produce a solid tumor leaving the left leg as a control in the peritoneal cavity to produce ascites tumor (Pressacco et al., 1994).

### 3.9 Biodistribution of ^125^I-coumarin derivative in normal mice

*In-vivo* biodistribution studies were performed using 4 groups each comprising six mice. Each animal was injected in the tail vein with 0.2 ml solution containing 5–10 kBq of radioiodinated coumarin derivative. The mice were kept in metabolic cages for the required time. Animals were subjected cervical dislocation at the recommended time (15 min, 30 min, 1 h or 4 h) after injection. Organs or tissues of interest were removed, washed with saline, weighed and counted. Correction was made for background radiation and physical decay during the experiment. The weights of blood, bone and muscles were assumed to be 7%, 10% and 40% of the total body weight, respectively (Mester et al., 1996).

### 3.10 Biodistribution of ^125^I-coumarin derivative in solid tumor bearing mice

A group of 24 solid tumor bearing mice was used for studying the biodistribution of the labeled drug at the selected times (15 min, 30 min, 1 h or 4 h) taking 6 mice at each time. Mice were sacrificed by cervical dislocation at various time intervals. Organs and tissues of interest were removed, washed, weighed and counted for its uptake of activity. Ascites fluid was drained and counted as a whole in a well type NaI (TI) gamma counter. The results were calculated as a percentage of injected dose (I.D) per gram tissue per body weight (Issidorides and Haddadin, 1996).

## 4 Results and discussion

**Fig. 2:**
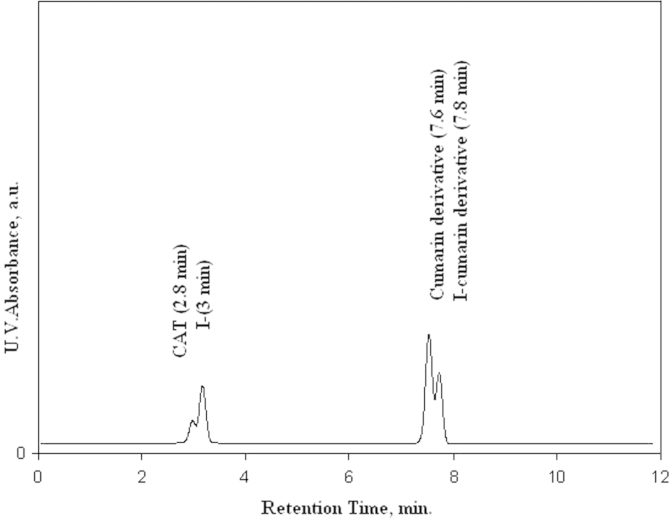
U.V. absorption chromatogram of the reaction mixture of substrate, CAT and KI.

**Fig. 3:**
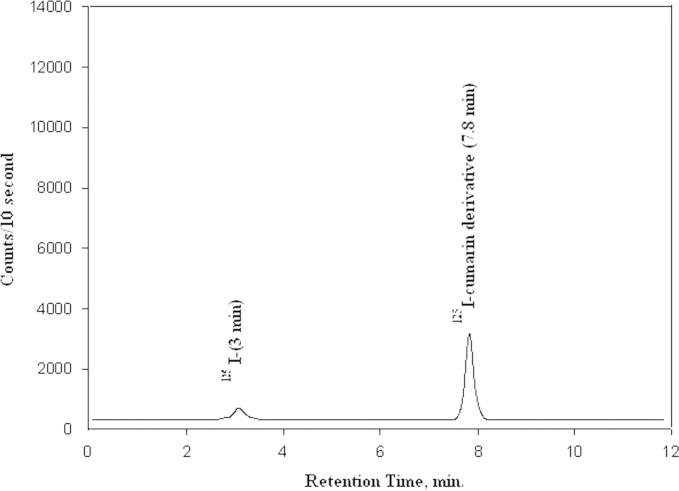
Radiochromatogram of the reaction mixture of substrate, CAT and Na^125^I.

HPLC analysis showed that the retention times of KI, coumarin derivative and ^125^I-coumarin derivative were 3.0, 7.6 and 7.8 min, respectively, identified by UV absorption as shown in Fig. 2. Fig. 3 shows that retention time of ^125^I^−^ and ^125^I-coumarin derivative was the same retention times of 3.0 and 7.8 min, respectively, as that of the inactive KI and iodo-compound detected by UV. HPLC analysis of ^125^I-coumarin complex was done by injection of 10 µl, after 0.22 µm Millipore filtration, into the column (RP-18. 300×3.9 mm 2, Alpha bond) and UV spectrophotometer detector (SPD-6A) adjusted to the 240 nm wavelength. The column was eluted with mobile phase (60% acetonitrile, 40% water), and the flow rate was adjusted to 1 ml/min. Then fractions of 1 ml were collected separately using a fraction collector up to 30 and counted in a well-type NaI (Tl) detector connected to a single-channel analyzer.

### 4.1 Electrophoresis analysis

**Fig. 4:**
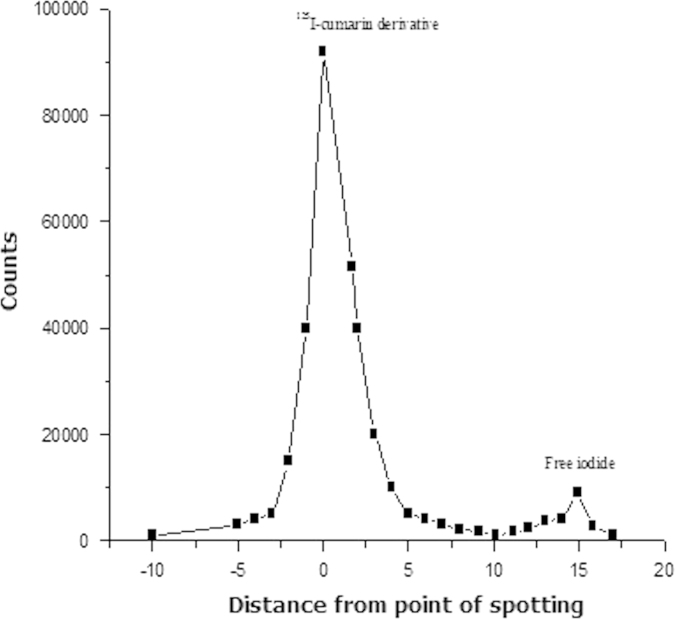
Paper electrophoresis pattern of the radioiodinated^125^I-coumarin.

Fig. 4 illustrates the analysis of the fractions that were produced from the reaction by electrophoresis. Two main peaks were formed, one corresponding to the free iodide that moved towards the anode with 16 cm distance at the condition mentioned before. The second peak which stayed near the point of spotting was found to be identical to that of ^125^I-iododeoxyuridine under the same electrophoretic conditions (Korde et al., 1998).

### 4.2 Factors affecting labeling yield

#### 4.2.1 Effect of oxidizing agents

**Fig. 5:**
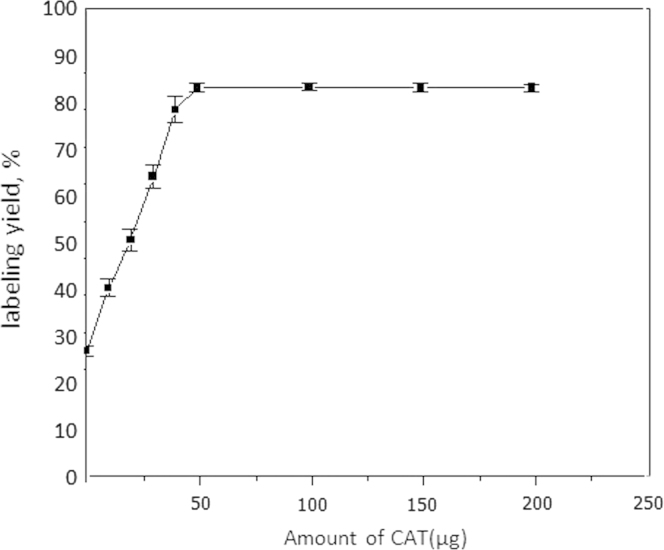
Variation of radiochemical yield of ^125^I-coumarin derivative as a function of chloramine-T amount as an oxidizing agent. Mean±SD (mean of three experiments). Reaction condition: 1 mg coumarin derivative, *X* µg chloramine-T, 10 µL Na^125^I. The reaction mixture was kept at 40 °C for 30 min.

Results obtained in this study revealed that the electrophilic substitution of the iodonium ion [^⁎^I^+^] onto coumarin derivative molecule afforded a high radiochemical yield by utilizing CAT as an oxidizing agent (Fig. 5). It was observed that the radiochemical yield significantly increased by increasing the amount of CAT from 10 μg to 50 μg (optimum content) at which maximum labeling yield was obtained. By increasing the amount of CAT above 50 μg, the yield showed no significant change. A significant decrease in the labeling yield was noted by decreasing the concentration of CAT below 50 μg that may be explained as at low concentrations of CAT, not all iodide converted to iodonium ion and thus, the yield was decreased (Walicka et al., 1998)

#### 4.2.2 Effect of substrate amount

**Fig. 6:**
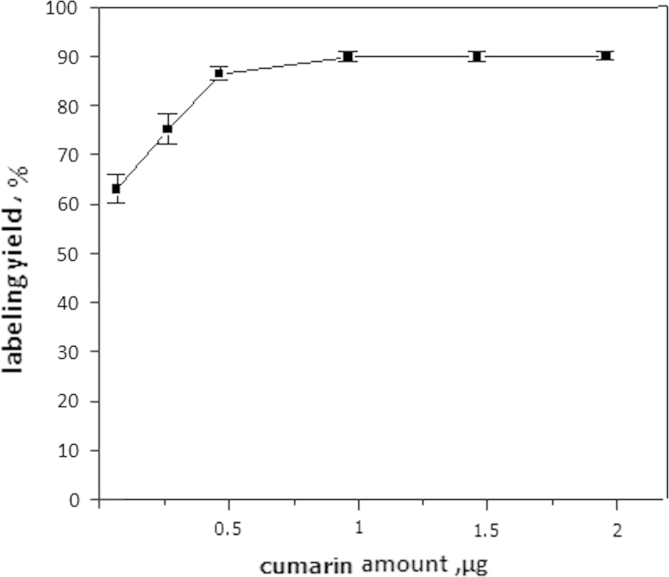
Variation of radiochemical yield of ^125^I-coumarin derivative with substrate (cumarin derivative) amount using chloramine-T as an oxidizing agent. Mean±SD (mean of three experiments). Reaction condition: *X* mg coumarin derivative, 50 µg chloramine-T, 10 µL Na^125^I. The reaction mixture was kept at 40 °C for 30 min.

The influence of coumarin derivative amount as a substrate on the labeling yield using CAT as an oxidizing agent is shown in Fig. 6. The increase of the amount of coumarin derivative was accompanied by a significant increase in the labeling yield, where it reached above 95% at 1 mg of coumarin derivative. Increasing the amount of coumarin derivative above 1 mg produced no significant increase in the labeling yield. Increasing the amount of starting material usually increases the total incorporation of radioiodine, since there is a minimum limit to the volume used (Wally et al., 2010). 1 mg of coumarin derivative was required to obtain maximum labeling yield, below this amount there was a significant decrease in the yield. On the other hand, using higher amounts did not significantly affect labeling yields.

#### 4.2.3 Effect of pH

As shown in Table 1, pH 7 is the optimum pH at which the maximum yield was obtained (97.3%). It was observed that at pH 4, the yield was 93.8% while at pH values 9 and 11 the yield was 93% and 81.7%, respectively. There was significant difference between all pH values of the reaction mediums.

**Table 1:** Effect of pH of the reaction medium on the labeling yield of ^125^I- coumarin.

pH value	% Labeled compound	% Free iodide
2	44.5±0.11	55.5±0.15
4	76.8±0.30⁎	23.2±0.2
7	93.8±0.30⁎	9.2±0.20
9	93.6±0.44⁎^,^†	6.4±0.25
11	81.2±0.20⁎^,^†	18.8±0.4

Values represent the mean±SEM *n*=6.

⁎ Significantly different from the initial values using unpaired student׳s *t*-test (*P*<0.05).

† Significantly different from the previous values using unpaired student׳s *t*-test (*P*<0.05).

#### 4.2.4 Effect of reaction time

**Fig. 7:**
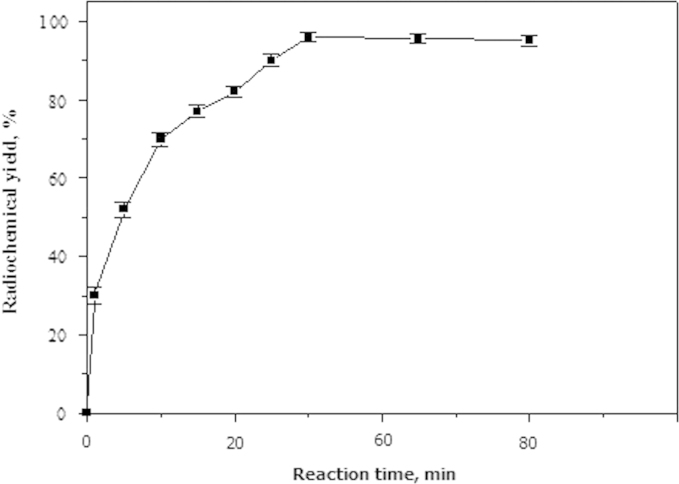
Variation of radiochemical yield of ^125^I-coumarin derivative with reaction time. Mean±SD (mean of three experiments). Reaction condition: 1 mg coumarin derivative, 50 µg chloramine-T, 10 µL Na^125^I. The reaction mixture was kept at 40 °C for *x* min.

Fig. 7 shows the relationship between the reaction time and the yield of ^125^I-coumarin derivative. Radiochemical yield was significantly increased from 30.9% to 94.8% with increasing reaction time from 1 min to 30 min. Extending the reaction time to 60 min produced no significant change of the radiochemical yield.

#### 4.2.5 Effect of reaction temperature

**Fig. 8:**
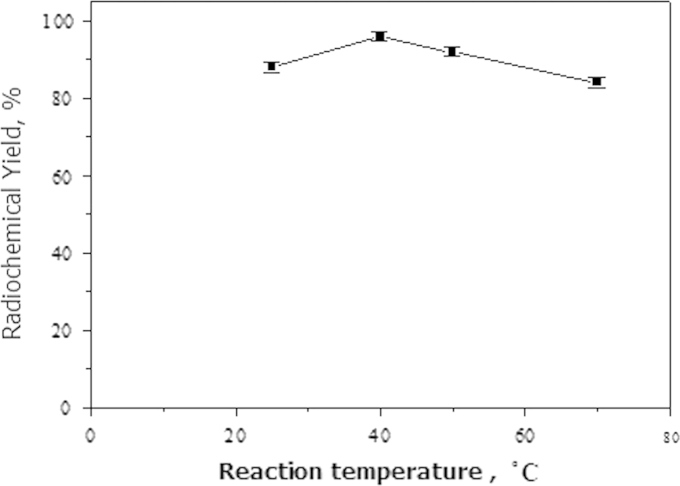
Variation of radiochemical yield of ^125^I-coumarin derivative with reaction temperature. Mean±SD (mean of three experiments). Reaction condition: 1 mg coumarin derivative, 50 µg chloramine-T, 10 µL Na^125^I. The reaction mixture was kept at *x* °C for 30 min.

The influence of reaction temperature on the radiochemical yield of ^125^I-coumarin derivative is shown in Fig. 8. The reactions were carried out at 25, 40, 50 and 70 °C. At room temperature, the labeling yield was 85±2.0% and increased to the maximum radiochemical yield 94.89±1.0% at 40 °C and decreased at higher temperature (70 °C) to 82.5±4.6%. This may be due to the thermal decomposition of the ^125^I-coumarin derivative.

### 4.3 *In-vitro* stability of ^125^I-coumarin derivative

In the present experiment, no significant change in the stability of ^125^I-coumarin derivative up to 48 h post-labeling is observed as shown in Table 2. This result showed the stability of the labeled compound.

**Table 2:** *In-vitro* stability of ^125^I-coumarin.

Time/hours	% Labeled compound	% Free iodide
1	94.1±0.36	6.9±0.55
6	93.7±0.30	7.3±0.2
12	93.9±0.40	6.1±0.1
24	92.8±0.30	8.2±0.2
48	92.5±0.30	7.5±0.30

Values represent the mean±SEM *n*=6.

### 4.4 Biodistribution of ^125^I-coumarin derivative

#### 4.4.1 In normal mice

A biodistribution study of ^125^I-coumarin derivative in normal mice showed that radioiodinated coumarin derivative was distributed rapidly in blood, stomach, kidney and liver at 15 min post-injection. After 30 min, ^125^I-coumarin derivative uptake was significantly decreased in organs like blood, liver and spleen. However, ^125^I-coumarin derivative uptake was significantly increased in stomach, bone, muscle, and thyroid after 30 min. At 1 h and 4 h post-injection, the majority of tissues showed significant decrease in ^125^I-coumarin derivative uptake. Thyroid gland showed significant increase in ^125^I-coumarin derivative uptake at 1 h post-injection as shown in Table 3.

**Table 3:** Biodistribution of ^125^I-coumarin in normal mice.

**Organs and body fluids**	**Percent I.D./gram organ**			
	**Time post-injection**			
	**15** **min**	**30** **min**	**1** **h**	**4** **h**
**Blood**	18.6±0.1	16.4 ±0.2⁎	8.4±0.04⁎	4.7±0.3⁎
**Bone**	1.3±0.05	1.9±0.10⁎	1.4±0.10⁎	0.7±0.1⁎
**Muscle**	1.1±0.01	1.7±0.02⁎	1.2±0.10	0.5±0.02⁎
**Liver**	4.4±0.05	1.8±0.15⁎	1.1±0.06⁎	1.0±0.02
**Lung**	2.7±0.1	5.10±0.12⁎	2.5±0.20⁎	1.2±0.01⁎
**Heart**	3.20±0.8	3.4±0.30⁎	2.0±0.01⁎	1.1±0.04⁎
**Stomach**	6.2±0.9	10.2±0.60*	9.6±0.16⁎	5.7±0.2⁎
**Intestine**	4.4±0.50	5.2±0.30⁎	3.5±0.1⁎	1.5±0.03⁎
**Kidney**	6.7±0.4	9.2±0.60⁎	3.1±0.3⁎	1.5±0.06⁎
**Urine**	6.5 ±0.7	15.5 ±0.7	23.5 ±0.7	36.5 ±0.7
**Spleen**	1.4±0.3	1.1±0.10⁎	1.0±0.02	0.8±0.05⁎
**Thyroid**	0.7±0.02	1.2±0.14⁎	4.1±0.16⁎	5.2±0.2

Values represent mean±SEM.

⁎ Means significantly differ from the previous each value using unpaired student׳s *t*-test (*p*<0.05).

#### 4.4.2 In solid tumor bearing mice

The sites of greatest uptake of ^125^I-coumarin derivative after 15 min post-injection were the blood, stomach, heart and lung, respectively. Table 4 shows that the concentration of ^125^I-coumarin derivative was the lowest in thyroid, spleen and muscle at 15 min post-injection. The uptake of ^125^I-coumarin derivative in solid tumor (right leg) was rapidly taking place as it received 3.5% of total activity. The uptake of solid tumor was significantly increased after 30 min and 1 h to reach 6.2% and 7.8% per g, respectively. No significant change in the uptake of ^125^I-coumarin derivative in solid tumor at 4 h post-injection was observed when compared to its previous value. The data also showed that some organs exhibit significant increase of uptake at 30 min post-injection like the stomach, solid tumor, kidney and thyroid. On the other hand, significant decrease in ^125^I-coumarin derivative uptake was observed in blood, heart and lung at the same time. At 1 h post-injection, the majority of organs showed significant decrease in uptake of ^125^I-coumarin derivative. Significant increase in ^125^I-coumarin uptake was only observed in solid tumor and thyroid at 1 h post-injection. Similarly, at 4 h post-injection, the majority of organs showed additional significant decrease in ^125^I-coumarin derivative uptake. The results revealed that the solid tumor was the site with one of the most uptake of ^125^I-coumarin derivative and this was seen at 30 min and lasted to 4 h post-injection. This result suggests the use of ^125^I-coumarin derivative in imaging of a tumor. The high uptake of ^125^I-coumarin derivative in the kidney may reflect the excretion of the drug *via* urine (Issidorides and Haddadin, 1996). The observation that the percentage of ^125^I-coumarin derivative concentration in the thyroid was significantly less than in other tissues indicates that less free iodide is associated with the ^125^I-coumarin derivative, since free iodide is rapidly captured by thyroid (Shriner, 1942). However, thyroid uptake was increased with time post-injection due to *in-vivo* deiodination of ^125^I-coumarin derivative (Ibrahim and Wally, 2010, Pressacco et al., 1994).

**Table 4:** Biodistribution of ^125^I-coumarin derivative in solid tumor bearing mice.

**Organs and body fluids**	**% injected dose/organ**			
	**Time post-injection**			
	**15** **min**	**30** **min**	**1** **h**	**4** **h**
**Blood**	19.3±1.8	10.81±0.6⁎	7.7±0.2⁎	3.2±0.2⁎
**Bone**	2.4 ±0.15	3.5±0.1	2.4±0.0⁎	1.1±0.1⁎
**Muscle**	1.3±0.01	1.9±0.02⁎	1.5±0.10	0.7±0.02⁎
**Liver**	4.5±0.4	7.7±0.2⁎	2.1±0.1⁎	1.7±0.1⁎
**Lung**	7.5±0.5	1.5±0.3⁎	4±0.2⁎	3.2±0.15⁎
**Heart**	9±0.4	4.4±0.1⁎	2.7±0.05⁎	2.5±0.2
**Stomach**	9.3 ±0.7	10.6±1⁎	7.5 ±0.5⁎	5.2±0.26⁎
**Intestine**	4.1±0.2	12.7±0.1⁎	14.1±0.3⁎	3.6±0.25
**Urine**	4.5±0.7	12.5 ±0.7	21.5 ±0.7	34.5 ±0.7
**Kidney**	2.5±0.7	6.6±0.2⁎	7.5±0.3⁎	2.5±0.2⁎
**Spleen**	0.7±0.5	1.2±0.4⁎	2.6±0.5⁎	1.4±0.05⁎
**Thyroid**	0.6±0.2	0.4±0.3⁎	2.1±0.5⁎	6.4±0.9⁎
**Left leg**	1.4±0.05	1.6±0.1	1.5±0.1	1.2±0.03⁎
**Right leg**	4.7±0.3	6.2 ±0.5⁎	7.8±0.2⁎	5.2±1.1⁎

Values represent mean±SEM *n*=6.

⁎ Significantly different from each previous value of each organ using unpaired student׳s *t*-test (*P*<0.05).

#### 4.4.3 Target/non-target ratio (T/NT)

Regarding the T/NT it was observed that it was more than 3, 3.8, 5 and 4.75 at 15 min, 30 min, 1 h and 4 h respectively. These values could provide the ability to image tumor sites (Table 5).

**Table 5:** T/NT values.

Time	15 m	30 m	1 h	4 h
T/NT	4.7/1.4	6.2/1.6	7.8/1.5	5.2/1.1

## 5 Conclusion

The incorporation of Auger emitters [^125^I] to a tumor site was achieved by labeling of coumarin derivative with iodine-125. The appropriate conditions for labeling of coumarin derivative (95% yield) were 50 μg CAT as oxidizing agent, 1 mg coumarin derivative as substrate at pH 7 at room temperature and 30 min reaction time. The great incorporation of ^125^I-coumarin derivative in tumor sites facilitates tumor imaging. ^125^I-coumarin derivative was found to be highly localized in tumor sites which considered an ideal vector to carry radioiodine to the nucleus of tumor cells and encourage further application to evaluate this radiolabeled compound *in-vivo* and *in-vi*tro on cancer cell lines. It also encourages the use of ^123^I-coumarin in the diagnosis of tumor sites.

## References

[bib1] Adelstein S.J., Kassis A.I. (1996). Acta Oncol..

[bib2] Al-Sehemi Abdullah G., El-Gogary Sameh R. (2012). Synthesis and photooxygenation of furo[3,2-c]coumarin derivatives as antibacterial and DNA intercalating agent animals. Chin. J. Chem..

[bib3] Brufola G., Fringuelli F., Piermatti O., Pizzo F. (1996). Heterocycles.

[bib4] Dall’Acqua F., Vedaldi D., Caffieri S., Guiotto A., Rodighiero P., Baccichetti F., Carlassare F., Bordin F. (1981). J. Med. Chem..

[bib5] Hara K., Sayama K., Ogha Y., Shinpo A., Suga S., Arakawa H. (2001). Chem. Commun..

[bib6] Ibrahim I.T., Wally M.A. (2010). J. Radioanal. Nucl. Chem..

[bib7] Issidorides C.H., Haddadin M.J. (1996). J. Org. Chem..

[bib8] Jiao C., Chen L., Shen G., Yu R. (2003). Sens. Actuators.

[bib9] Johnson J.R. (1942). Org. React..

[bib10] Jones G. (1967). Org. React..

[bib11] Kassis AI, Kirichian A.M., Wang K., Semnani E.S., Adelstein S.J. (2004). Int. J. Radiat. Biol..

[bib12] Kassis A.I. (2003). J. Nucl. Med..

[bib13] Korde A., Venkatesh M., Sarma H.D.,1998. In: Procedings of the International Symposium on Modern Trends in Radiopharmaceuticals for Diagnosis and Therapy, IAEA, Lisbon, Portugal, March 30–April 3, SM-355/13.

[bib14] Maria J.B., Santos C., Susana N., Barbara G., Mario N.B.S. (2004). Tetrahedron Lett..

[bib15] Mester J., DeGoeij K., Sluyser M. (1996). Eur. J. Cancer.

[bib16] Murray R.D.H., Mendez J., Brown S.A. (1982). The Natural Coumarins: Occurrence, Chemistry and Biochemistry.

[bib17] O’Kennedy R., Thornes R.D. (1997). Coumarins: Biology, Applications and Mode of Action.

[bib18] Pressacco J., Hedley D.W., Erlichman C. (1994). Cancer Res..

[bib19] Shriner R.L. (1942). Org. React..

[bib20] Singer L.A., Long N.P. (1996). J. Am. Chem. Soc..

[bib21] von Pechmann H., Duisberg C. (1884). Chem. Ber..

[bib22] Walicka M.A., Adelstein S.J., Kassis A.I. (1998). Radiat. Res..

[bib23] Wally M.A., El-Gogray S.R., El-Sepelgy O.Z. (2010). Synth. Comm..

[bib24] Yavari I., Hekmat-Shoar R., Zonouzi A. (1998). Tetrahedron Lett..

[bib25] Zahradnik M. (1992). The Production and Application of Flourescent Brightening Agents.

